# Layered Birnessite Cathode with a Displacement/Intercalation Mechanism for High-Performance Aqueous Zinc-Ion Batteries

**DOI:** 10.1007/s40820-020-0397-3

**Published:** 2020-02-18

**Authors:** Xian-Zhi Zhai, Jin Qu, Shu-Meng Hao, Ya-Qiong Jing, Wei Chang, Juan Wang, Wei Li, Yasmine Abdelkrim, Hongfu Yuan, Zhong-Zhen Yu

**Affiliations:** 1 State Key Laboratory of Organic-Inorganic Composites, College of Materials Science and Engineering, Beijing University of Chemical Technology, Beijing, 100029 People’s Republic of China; 2 Beijing Key Laboratory of Advanced Functional Polymer Composites, Beijing University of Chemical Technology, Beijing, 100029 People’s Republic of China

**Keywords:** Zinc-ion batteries, Birnessite, Sodium ions, Layered structure, Crystal water

## Abstract

Mn-based rechargeable aqueous zinc-ion batteries (ZIBs) are highly promising because of their high operating voltages, attractive energy densities, and eco-friendliness. However, the electrochemical performances of Mn-based cathodes usually suffer from their serious structure transformation upon charge/discharge cycling. Herein, we report a layered sodium-ion/crystal water co-intercalated Birnessite cathode with the formula of Na_0.55_Mn_2_O_4_·0.57H_2_O (NMOH) for high-performance aqueous ZIBs. A displacement/intercalation electrochemical mechanism was confirmed in the Mn-based cathode for the first time. Na^+^ and crystal water enlarge the interlayer distance to enhance the insertion of Zn^2+^, and some sodium ions are replaced with Zn^2+^ in the first cycle to further stabilize the layered structure for subsequent reversible Zn^2+^/H^+^ insertion/extraction, resulting in exceptional specific capacities and satisfactory structural stabilities. Additionally, a pseudo-capacitance derived from the surface-adsorbed Na^+^ also contributes to the electrochemical performances. The NMOH cathode not only delivers high reversible capacities of 389.8 and 87.1 mA h g^−1^ at current densities of 200 and 1500 mA g^−1^, respectively, but also maintains a good long-cycling performance of 201.6 mA h g^−1^ at a high current density of 500 mA g^−1^ after 400 cycles, which makes the NMOH cathode competitive for practical applications.

**Electronic supplementary material:**

The online version of this article (10.1007/s40820-020-0397-3) contains supplementary material, which is available to authorized users.

## Highlights

- A layered sodium-ion/crystal water co-intercalated Na_0.55_Mn_2_O_4_·0.57H_2_O (NMOH) cathode is synthesized successfully with a selectively etching method for zinc-ion batteries.
- A displacement/intercalation mechanism is confirmed in the Mn-based cathode for the first time.
- The NMOH cathode delivers a competitive reversible capacity of 201.6 mA h g^−1^ at 500 mA g^−1^ after 400 cycles.

**Electronic supplementary material:**

The online version of this article (10.1007/s40820-020-0397-3) contains supplementary material, which is available to authorized users.

## Graphical abstract

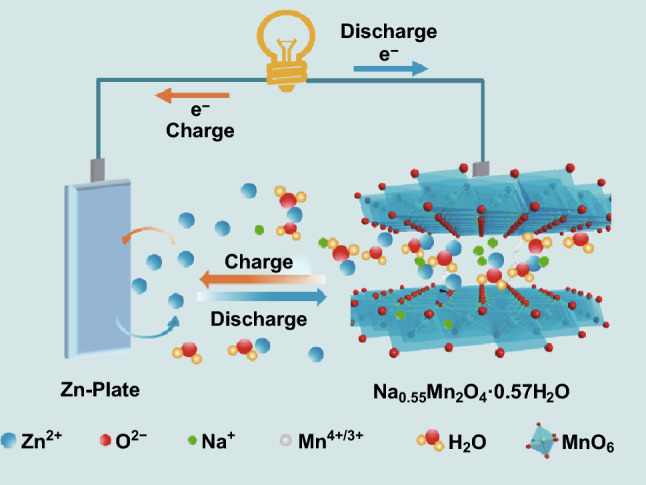

## Introduction

Eco-friendly energy storage technologies continue to attract wide attentions due to the serious environmental pollution and energy consumption [1–8]. Rechargeable aqueous metal-ion (Li^+^, Na^+^, Zn^2+^, Mg^2+^, Ca^2+^, etc.) batteries exhibit considerable advantages for grid-scale energy storage technologies, due to their eco-friendliness, low-cost, and 100–1000 times higher ionic conductivities of aqueous electrolytes than those of organic electrolytes [1, 9–12]. Recently, much effort has been devoted to zinc-ion batteries (ZIBs) because of their high safety with aqueous electrolytes, high abundance of zinc element, and the low redox potential of Zn/Zn^2+^ at − 0.76 V vs SHE [13–15]. Various materials of Prussian blue analogues (PBAs), V-based compounds, and Mn-based materials have been utilized as cathodes for zinc-ion storage. PBAs have high output operation voltages, but their low capacities hinder their further developments [16, 17]. Although layered V-based compounds, such as VS_2_ [18], H_2_V_3_O_8_ [19], and Mg_*x*_V_2_O_5_·*n*H_2_O [20], exhibit presentable electrochemical performances for aqueous ZIBs, the low operation voltage, high cost, and high toxicity of vanadium impede their wide applications [21]. Given the high theoretical capacities, low-cost, and high-voltage platforms, Mn-based materials are competitive for high-performance cathodes of ZIBs. Various Mn-based materials have been reported, such as α- [22, 23], β- [24], γ-phase [25], and other types [26–28] of manganese dioxide, spinel-type ZnMn_2_O_4_ [14, 29, 30], and MgMn_2_O_4_ [31]. However, the development of the Mn-based cathode materials is limited by low specific capacity or poor cyclability, ascribing to the irreversible phase transformation. Therefore, it is still on the way to find suitable Mn-based cathodes.

The reported Mn-based cathodes usually have two types of crystal structures: tunnel structure and layered structure. The tunnel structure is less favor of intercalation and diffusion of zinc ions than the layered structure on the basis of zinc-ion insertion/extraction mechanisms [20], so layered Mn-based cathodes would be more beneficial for aqueous ZIBs. For example, Qian et al. [32] reported an aqueous ZIB with a layered δ-MnO_2_ cathode, exhibiting a specific capacity of 133 mA h g^−1^ at the current density of 100 mA g^−1^. Kim et al. [33] also fabricated a cell with a δ-MnO_2_ cathode, presenting a discharge capacity of 250 mA h g^−1^ at a low current density of 83 mA g^−1^ with a high Coulombic efficiency close to 100%. However, a drawback of the layered δ-MnO_2_ is its irreversible phase transformation to other phases (e.g., ZnO, MnO, and MnOOH), causing a significantly volume change of the active materials and thus resulting in capacity decay and low specific capacity. Besides, the tremendous electrostatic interaction between zinc ions and host materials usually leads to sluggish diffusion of zinc ions, limiting the development of ZIBs [19]. Fortunately, alkali metal ions of Na^+^, K^+^, and Li^+^, and crystal water could serve as pillars to stabilize the layered structure and improve the interlayer distance and, hence, benefit the diffusion of guest ions in electrode materials [34–39]. For example, Mai et al. [40] demonstrated a Na_0.33_V_2_O_5_ cathode, in which the sodium ions stabilized the layered structure and improved the electronic conductivity for high-performance ZIBs. However, the role of alkali metal ions or crystal water in Mn-based cathode of ZIBs has been rarely studied.

Herein, layered sodium-ion/crystal water co-intercalated Birnessite with the formula of Na_0.55_Mn_2_O_4_·0.57H_2_O (NMOH) is fabricated by rearranging MnO_6_ octahedrons with sodium ions and water molecules via selectively etching the silica tetrahedrons of manganese silicate with NaOH solution. The resultant NMOH is used as the cathode material of aqueous ZIBs. As a Mn-based cathode, the NMOH is subjected to a displacement/intercalation electrochemical mechanism: Sodium ions and crystal water could enlarge the interlayer distance to speed up the transport of zinc ions, and the partial substitution of Na^+^ with Zn^2+^ in the first cycle could support the layered structure, facilitating subsequent reversible Zn^2+^/H^+^ insertion/extraction. Additionally, the surface-adsorbed sodium ions also contribute to the pseudo-capacitance. As a result, the NMOH electrode of ZIBs exhibits a high specific capacity of 389.8 mA h g^−1^ at a current density of 200 mA g^−1^, and a satisfactory long-term cycle performance with a high capacity of 201.6 mA h g^−1^ at 500 mA g^−1^ after 400 cycles. The roles of sodium ions and crystal water in the Mn-based cathode of ZIBs, and the synergy between sodium ions and crystal water are studied in detail. The replacement/intercalation reaction mechanism is also elucidated.

## Experimental Section

### Materials

Zinc sulfate (ZnSO_4_·H_2_O, 99.9%), sodium sulfate (Na_2_SO_4_, 99.9%), and manganese sulfate (MnSO_4_·H_2_O, 99.9%) were purchased from Aladdin (China). Sodium silicate (Na_2_SiO_3_·9H_2_O) was obtained from Xilong Scientific Co., Ltd (China). Zn foil (99.98%) and titanium foil (99.8%) were provided by Alfar Aesar Chemicals (China). Manganese chloride tetrahydrate (MnCl_2_·4H_2_O, 99%), sodium hydroxide (NaOH), and potassium permanganate (KMnO_4_) were supplied by Beijing Chemical Factory (China). All the chemicals were used as received.

### Synthesis of Mn_*x*_Si_*y*_O_*z*_

In a typical synthesis of Mn_*x*_Si_*y*_O_*z*_ (MSO), 3.2 mmol of sodium silicate was dissolved in 40 mL of deionized (DI) water as solution A, while 3.2 mmol of manganese dichloride tetrahydrate was dissolved in 60 mL of deionized water as solution B. After mixing the two solutions under stirring for 60 min at room temperature, the resultant MSO was centrifuged, washed thoroughly using DI water and ethanol several times, and dried at 80 °C for 10 h in air.

### Synthesis of Na_0.55_Mn_2_O_4_·0.57H_2_O

Typically, the synthesized Mn_*x*_Si_*y*_O_*z*_ was dispersed in 2 M of sodium hydroxide solution, and the suspension was reacted under stirring in a water bath at 90 °C for 12 h to fabricate Na_0.55_Mn_2_O_4_·0.57H_2_O (NMOH). The resulting NMOH was collected by centrifugation, washed with deionized water and ethanol for several times, and dried at 60 °C for 12 h.

### Synthesis of δ-MnO_2_

To synthesize the δ-MnO_2_ powder, the KMnO_4_ was annealed at 350 °C for 5 h with a heating speed of 5 °C min^−1^ under an argon atmosphere [33]. Then, the solid resultant was washed with deionized water, then collected by centrifugation, and finally dried at 60 °C for 12 h.

### Characterization

Microstructures of MSO, NMOH, and electrode were analyzed using transmission electron microscopy (TEM, JEOL JEM-1011), scanning electron microscopy (SEM, Hitachi S4700), and high-resolution TEM (HRTEM, JEOL JEM-3010). Crystal structures of active materials were investigated with a Rigaku D/Max 2500 X-ray diffractometer (XRD) using a Cu Kα radiation. Compositions of the NMOH were measured by a Thermo VG RSCAKAB 250 high-resolution X-ray photoelectron spectroscopy (XPS). Thermal stability of NMOH was carried out on a TA Q50 thermogravimetric analyzer (TGA) at a heating speed of 10 °C min^−1^ under an argon atmosphere. Specific surface area was measured based on N_2_ adsorption/desorption isotherms with a JW-BK132F (JWGB SCI & TECH) analyzer. Inductively coupled plasma atomic emission spectroscopy (ICP-AES) was performed on ICAP6300 Radial. For the ex situ XRD measurements, the cathodes obtained at specific voltages were washed thoroughly with distilled water and ethanol and these electrodes were then dried at 60 °C for 10 h in vacuum.

### Electrochemical Characterization

Electrochemical measurements of the NMOH were performed with CR2032 coin-type cells assembled in air. To fabricate working electrode, the NMOH, super-P, RGO, and poly(vinylidene fluoride) with a mass ratio of 70:15:5:10 were mixed by ball milling with *N*-methyl-2-pyrrolidone as the solvent. The obtained slurry was coated onto a Ti foil and vacuum-dried at 80 °C for 12 h. The loading mass of the active material was ~ 1.0–1.5 mg cm^−2^. Zinc foil and glass fiber membrane were used as the anode and separator, respectively. A 2 M ZnSO_4_ with/without 0.2 M MnSO_4_ aqueous solution was used as the electrolyte. The charge/discharge experiments were performed on a Land BT2000 battery test system (China) at room temperature. Cyclic voltammetry (CV) and electrochemical impedance spectroscopy (EIS, 100 kHz to 0. 01 Hz) were obtained on a CHI 760E electrochemical workstation.

## Results and Discussion

**Fig. 1:**
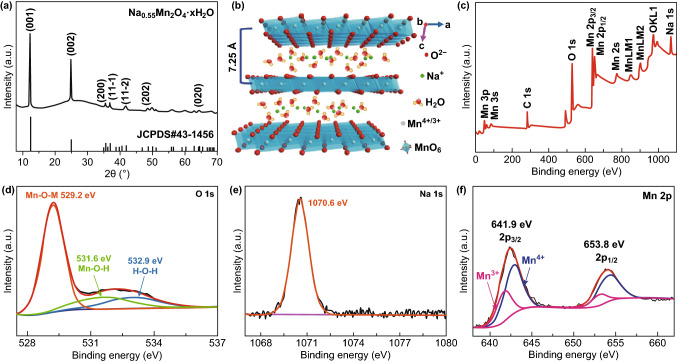
**a** XRD pattern of NMOH. **b** Illustration of crystal structure of Na_0.55_Mn_2_O_4_·*x*H_2_O. XPS spectra of NMOH: **c** survey scan, **d** O 1*s* spectrum, **e** Na 1*s* spectrum, and **f** Mn 2*p* spectrum

Figure 1a shows XRD pattern of the as-synthesized NMOH. Different from the amorphous phase of Mn_*x*_Si_*y*_O_*z*_ (MSO) with a broad peak (Fig. S1a), the NMOH has obvious sharp peaks, belonging to high-purity layered Na-rich Birnessite of Na_0.55_Mn_2_O_4_·*x*H_2_O (monoclinic phase, JCPDS No. 43-1456) with the C2/m space group [41, 42]. As illustrated in Fig. 1b, the layered structure is constructed by the sheets consisting of two-dimensional edge-sharing MnO_6_ octahedrons, in which Mn and O atoms occupy 2a and 4i sites, respectively [42, 43]. The interlayer spacing of the layered NMOH determined from its (001) plane at 12.3° is 7.25 Å. Many sodium ions do exist with significant amounts of crystal water in the intra-gallery to stabilize the layered structure and expand the space between the (001) planes, which could facilitate the transport and diffusion of zinc ions during the charge/discharge process, inhibit the manganese ion dissolution caused by the Jahn–Teller effect [31, 37, 38], and improve the performances of the ZIBs.

The δ-MnO_2_ particles (monoclinic phase, JCPDS No. 42-1317) with the layered structure are fabricated by the thermal decomposition of KMnO_4_ (Fig. S1b) [33, 43] and exhibit an irregular morphology (Fig. S1c). The XRD pattern (Fig. S1d) unveils that the interlayer distance of NMOH is larger than that of the δ-MnO_2_, resulting from the presence of Na^+^ and crystal water between the adjacent layers of NMOH. Figure S2a shows TGA curve of NMOH measured under an argon atmosphere from room temperature up to 1000 °C. The mass loss in the range of 27–100 °C derives from the removal of surface free water, while the mass loss of 5.24 wt% from 100 to 300 °C is assigned to the removal of crystal water. Therefore, the *x* value in Na_0.55_Mn_2_O_4_·*x*H_2_O could be calculated as ~ 0.57.

To explore the compositions and valence states of the NMOH, Fig. 1c presents the XPS survey spectrum of NMOH, showing the peaks of Na, Mn, and O. The high-resolution O 1*s* spectrum (Fig. 1d) displays three fitting peaks at 529.2, 531.6, and 532.9 eV corresponding to Mn–O–M, Mn–O–H, and H–O–H bonds, respectively [23, 44]. The presence of sodium ions is further verified by the high-resolution Na 1*s* spectrum with the binding energy of 1070.6 eV (Fig. 1e) [41, 45]. The two peaks of Mn 2*p* (Fig. 1f) located at 641.9 and 653.8 eV are attributed to Mn 2*p*_3/2_ and Mn 2*p*_1/2_, respectively [41, 44]. Additionally, the four fitting peaks indicate that Mn^4+^ and Mn^3+^ ions are dominant in the NMOH to keep the electrical neutrality, because of the presence of sodium ions. The coexistence of Mn^4+^ and Mn^3+^ is also consistent with the average valence of Mn in the NMOH (ca. + 3.73). All these results confirm the successful fabrication of layered sodium-ion/crystal water co-intercalated Na_0.55_Mn_2_O_4_·0.57H_2_O.

**Fig. 2:**
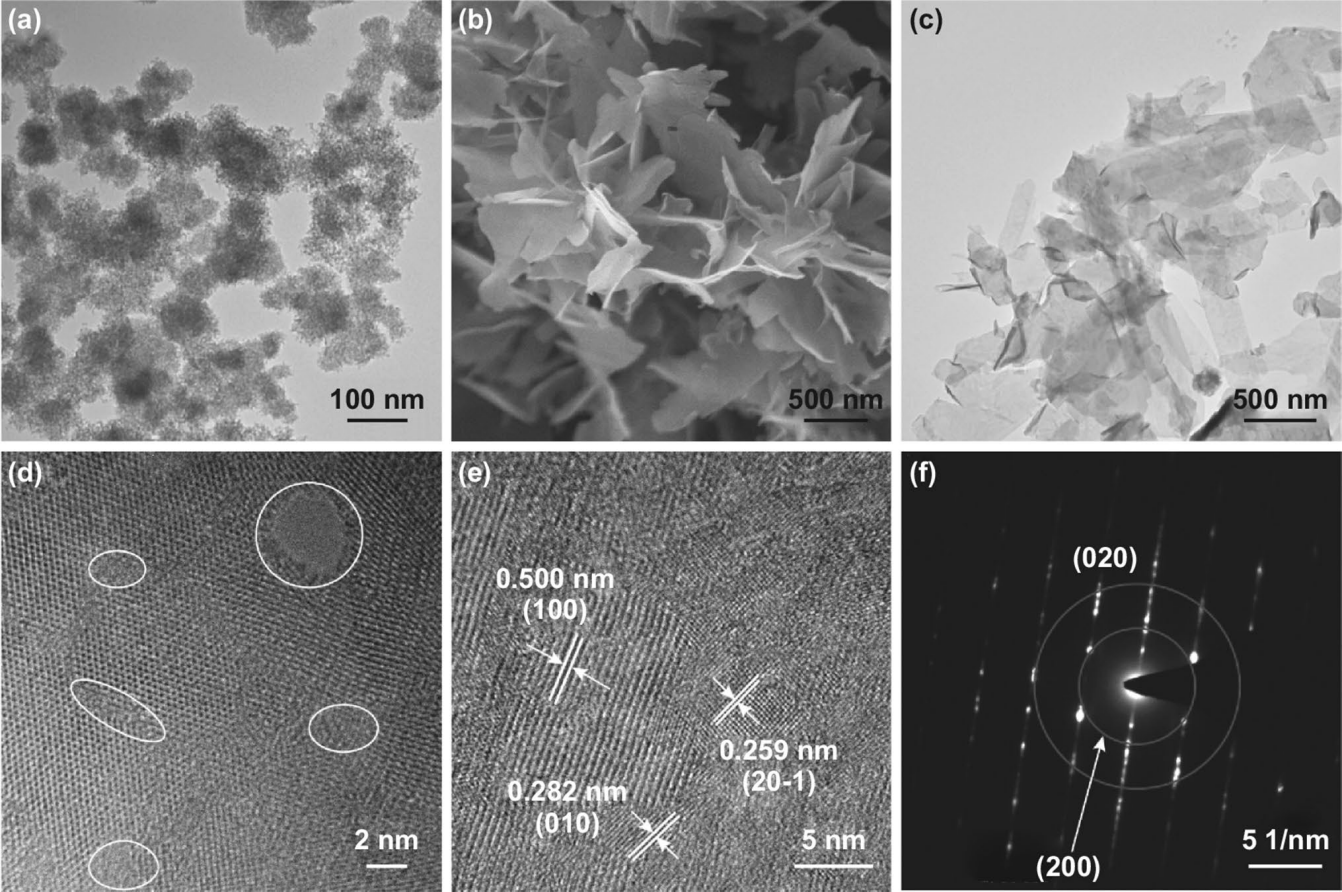
**a** TEM image of Mn_*x*_Si_*y*_O_*z*_. **b** SEM image, **c** TEM image, **d, e** HRTEM images, and **f** corresponding SAED pattern of Na_0.55_Mn_2_O_4_·0.57H_2_O

Figure 2a shows the TEM image of MSO. Apparently, the MSO is composed of many worm-like nanoparticles. As a typical metal silicate, the crystal structure of MSO should contain MnO_6_ octahedrons and silica tetrahedrons [46, 47]. During the etching treatment, silica tetrahedrons are selectively removed by the NaOH solution, while the residual MnO_6_ octahedrons rearrange with sodium ions and water molecules to form NMOH nanosheets with smooth surface (Fig. 2b). Some black nanoparticles are formed within the etching time of 9 h (Fig. S1e–h). XRD patterns of the NMOH with different etching time show that these black nanoparticles should be indexed to the phase of Mn_3_O_4_ (Fig. S1a). However, these black nanoparticles would completely disappear after etching twelve hours to form pure NMOH nanosheets (Fig. S1i, j). The N_2_ adsorption–desorption analysis is conducted to identify the BET specific surface area of the NMOH and δ-MnO_2_ (Fig. S2b). The layered NMOH has a higher specific surface area (81.6 m^2^ g^−1^) than that of δ-MnO_2_ (13.5 m^2^ g^−1^). Meanwhile, abundant mesopores with a relatively narrow pore-size distribution of ~ 8 nm provide a large number of active sites for storage of zinc ions and enhance the contact area of the active material with the electrolyte, promoting the transport and diffusion of zinc ions [41, 48, 49]. In addition, the presence of these mesopores is favorable for regulating the volume change caused by the insertion/extraction of zinc ions during the discharge/charge process, ensuring a satisfactory cyclability and a high specific capacity [3, 50].

TEM image of NMOH (Fig. 2c) shows typical nanosheets with length and width of approximately 100–500 nm, which is consistent with its SEM results. The EDS mapping reveals that the Na, Mn, and O elements are uniformly distributed on the NMOH nanosheets (Fig. S3). The plenty crystal boundaries and mesopores displayed by the white circles (Fig. 2d) could facilitate the insertion/extraction of zinc ions from the edge. Besides, the clear lattice spacings of 0.500, 0.282, and 0.259 nm match well with the inter-planar distances of the (100), (010), and (20-1) planes, respectively (Fig. 2e). The corresponding SAED pattern also confirms the polycrystalline nature of the NMOH (Fig. 2f). These results verify the mesoporous and layered structure of the NMOH nanosheets.

**Fig. 3:**
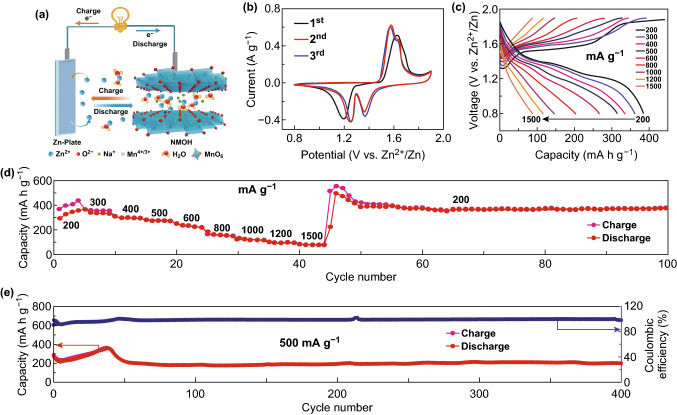
Electrochemical performances of NMOH electrode. **a** Working principle of an aqueous ZIB with NMOH as the cathode and Zn foil as the anode. **b** CV curves of coin-type Zn/NMOH cell with the aqueous electrolyte of 2 M ZnSO_4_ and 0.2 M MnSO_4_ at a scan rate of 0.1 mV s^−1^. **c** Galvanostatic charge/discharge profiles of the Zn/NMOH cell tested between 0.8 and 1.9 V with different current densities. **d** Rate performances of NMOH electrode at current densities between 200 and 1500 mA g^−1^. **e** Long-cycling performances of NMOH electrode at 500 mA g^−1^

The zinc-ion storage performances of NMOH nanosheets in an aqueous electrolyte (2 M ZnSO_4_ + 0.2 M MnSO_4_) are investigated using the CR2032 coin-type cell with NMOH as the cathode and Zn foil as the anode (Fig. 3a). Figure 3b shows the first three CV curves of the NMOH cathode performed at the scan rate of 0.1 mV s^−1^ within the voltage window of 0.8–1.9 V (vs. Zn/Zn^2+^). In the first cathodic cycle, the peak at 1.19 V corresponds to the electrochemical insertion of zinc ions into the NMOH nanosheets. A broad peak centered at 1.62 V with a shoulder peak at 1.68 V can be observed in the first anodic cycle. These two peaks are mainly caused by the extraction of zinc ions and sodium ions out of the intra-gallery of NMOH nanosheets and the oxidation of manganese ions. After the first cycle, the two well-defined cathodic peaks at 1.24 and 1.36 V, and the two anodic peaks at 1.57 and 1.62 V are nearly the same in the subsequent second and third CV curves, corresponding to a two-step electrochemical reaction with an excellent reversibility, consistent with the galvanostatic charge/discharge curves (Fig. S4). The rate and cycling performances of the NMOH cathode are measured at different current densities from 200 to 1500 mA g^−1^ in the aqueous electrolyte of 2 M ZnSO_4_ with 0.2 M MnSO_4_. Figure 3c presents the galvanostatic charge/discharge curves of the NMOH cathode at varied current densities. The NMOH electrode exhibits excellent rate performances with average reversible capacities of 340.7, 350.2, 307.8, 285.2, 237.1, 165.7, 128.6, 102.1, and 87.1 mA h g^−1^ at current densities of 200, 300, 400, 500, 600, 800, 1000, 1200, and 1500 mA g^−1^, respectively (Fig. 3d). When the current density returns to 200 mA g^−1^, the specific capacity of the NMOH electrode is fully recovered and even slightly higher than the initial value, indicating the excellent electrochemical performance. The NMOH cathode continues to conduct the cycling performance at the current density of 200 mA g^−1^. After 100 cycles, the specific capacity is still stable at 389.8 mA h g^−1^, demonstrating the good stability and reversibility of the NMOH cathode.

Besides, the electrochemical performances of the coin-type Zn/NMOH cell using an aqueous electrolyte of 2 M ZnSO_4_ without the 0.2 M MnSO_4_ additive are also explored. The overpotential of the cell in the bare ZnSO_4_ electrolyte is higher than that in the ZnSO_4_ electrolyte with MnSO_4_ (Fig. S5a), and the specific capacity decreases rapidly to lower than 100 mA h g^−1^ just after several cycles in the electrolyte without the MnSO_4_ additive at the current density of 200 mA g^−1^ (Fig. S5b), indicating the important role of MnSO_4_ in the charge/discharge processes. It is quite common that the structure of Mn-based electrode materials could be destroyed during the cycling, which is caused by the dissolution of Mn^2+^ from Mn^3+^ disproportionation due to the Jahn–Teller distortion [13, 30]. In the present work, MnSO_4_ could prevent the dissolution of manganese from the NMOH, resulting in a higher specific capacity. Figure S6 shows SEM image and corresponding EDS spectrum of the NMOH electrode after recharging to 1.9 V. The molar ratio of O/Mn (~ 2.465) is nearly consistent with the original O/Mn state (~ 2.285), revealing the excellent stability of the NMOH nanosheets in the presence of the MnSO_4_ additive. Therefore, the NMOH electrode exhibits a high Coulombic efficiency nearly up to 100% with a steady specific capacity of 201.6 mA h g^−1^ for at least 400 cycles at the current density of 500 mA g^−1^ (Fig. 3e). The corresponding charge/discharge curves at 10th, 300th, and 400th cycles show similar voltage profiles (Fig. S7), indicating the excellent stability of zinc-ion egress/ingress in the NMOH nanosheets. Besides, the obtained specific capacities of NMOH at varied current densities are higher than those of Mn-based aqueous ZIBs reported recently (Table S1), confirming the advantage of the layered sodium-ion/crystal water co-intercalated Birnessite cathode.

To confirm the crucial roles of Na^+^ and crystal water, the electrochemical performances of neat δ-MnO_2_ and annealed NMOH electrodes are also examined under the same conditions. Neat δ-MnO_2_ exhibits a similar layered structure to NMOH, but it is not stabilized with Na-ions. The layered structure is favorable for the diffusion of Zn^2+^, thus the δ-MnO_2_ electrode could perform a high discharge capacity of 194.2 mA h g^−1^ at 500 mA g^−1^ in the second cycle, but the capacity dropped swiftly to 72.0 mA h g^−1^ after 40 cycles (Fig. S8), strongly supporting the crucial role of Na^+^ in the Zn^2+^ storage of the novel NMOH cathode. To confirm the considerable role of the crystal water content in the Zn^2+^ storage performance, the NMOH samples are thermally annealed at 150 and 300 °C for 2 h in Ar, which are designated as NMOH-150 and NMOH-300, respectively. The content of crystal water decreases with the increasing temperature (Fig. S9a). More critically, the two NMOH samples still maintain their layered structure due to stabilization effect of the sodium ions, but the intensities of the (001) and (002) peaks evidently decrease due to the removal of crystal water after the heat treatment (Fig. S9b). Besides, the interlayer space decreases with increasing the temperature, as confirmed by the shifts of the (001) and (002) peaks (Fig. S9b). As shown in Fig. S10, the electrochemical performances become worse with the decreasing crystal water content. Clearly, both Na^+^ and crystal water indeed play important roles in stabilizing the layered structure and enhancing the electrochemical properties of the NMOH electrode.

**Fig. 4:**
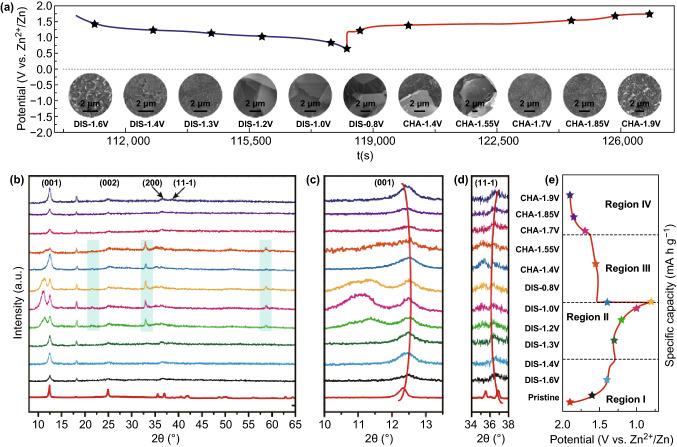
**a** Typical charge/discharge profile and corresponding SEM images of NMOH cathode in the second cycle. **b**–**e** Ex situ XRD patterns at different charge/discharge states and corresponding GCD curves of NMOH cathode at the current density of 200 mA g^−1^ in the second cycle

Interestingly, an activation process is observed in the long-term stability tests (Fig. 3e). Ex situ SEM images of the dissembled cells are used to investigate changes in surface morphologies. After the first electrochemical cycle, the surface of the electrode becomes rough (Fig. 4a), benefiting from the in situ redeposition of MnO_*x*_ derived from the dissolved manganese ions [30, 51]. The wettability of the electrode increases gradually upon cycling, leading to a faster ion transport efficiency, thus improving the performances of the ZIB, which is also a vital reason for the initial activation process [34, 41]. In addition, the formation of defects caused by the dissolution of Mn upon discharge leads to enriched active sites on the electrode surface, benefiting the improvement in the zinc-ion insert/extract reaction kinetics [41, 52]. The slight capacity fading after the activation process is also observed in previous studies. In the present work, the decrease in capacity may be caused by the continuous consumption of electrolyte or the impermeable passivation layer formation on the Zn anode [30, 31, 33, 34, 43, 52]. Additionally, the deposition of Zn_4_SO_4_ (OH)_6_·0.5H_2_O flakes on the surface of Zn anode after a long cycling (Fig. S11) may reduce the ion transport rate of zinc ions and increase the interfacial impedance of the cell, thus leading to the fading process. Then, a relative high reversible capacity could be maintained at 201.6 mA h g^−1^ for the subsequent cycles.

The reaction mechanism of zinc-ion storage is further evaluated using ex situ XRD, SEM, EDS, and XPS at various discharge/charge states during the second cycle within the potential window of 0.8–1.9 V at 200 mA g^−1^, and the corresponding discharge/charge states are marked in the charge/discharge profile (Fig. 4e). As depicted in Fig. 4b, the ex situ XRD patterns demonstrate the peak changes of the NMOH electrode. The prominent diffraction peaks of NMOH are observed at different discharge and charge states, indicating that the layered structure of NMOH is retained during the zinc-ion insertion/extraction processes. In detail, during the discharging process, the peak of (001) shifts gradually from 12.3° toward a higher angle (Fig. 4c), while the peak of (11-1) shifts toward a lower angle (Fig. 4d).The peak shifts of (001) imply a reduction in the interlayer distance of the NMOH during the discharge process (Fig. 4c), because of the strong electrostatic interaction between the MnO_6_ octahedral sheets and the intercalated zinc ions [20, 43]. The peak shifts of (11-1) demonstrate an increase in the inter-planar distance during the discharge process (Fig. 4d), which is caused by the expansion of the Mn–O bonds upon Mn^4+^/Mn^3+^ and Mn^4+^/Mn^2+^ reductions [43], since the bond lengths of the Mn^2+^-O and Mn^3+^-O are longer than that of Mn^4+^-O [53]. Interestingly, the (001) and (11-1) peaks could return to their original positions after the charging reaction, implying that the intercalation/deintercalation of zinc ions is a reversible process without the destruction of the NMOH.

Further analyses of the ex situ XRD show that no new significant diffraction peaks appear at the Region I (the first discharge platform, Fig. 4e), while multiple new peaks emerge at the Region II (the second discharge platform, Fig. 4e). The peak, which appears at 10.5° and disappears at the beginning of charging, might be assigned to the extra water layer, due to the solvation effect in the form of the water shell around the zinc ions during the discharging process. As reported, the water shell around the zinc ions could reduce the electrostatic interaction between the zinc ions and the MnO_6_ octahedral sheets to increase the ion transport rate [18, 19, 38]. Besides, the emerging peaks at 21.5°, 32.9°, and 58.7° in the Region II correspond to the new phase of zinc sulfate hydroxide hydrate (Zn_4_SO_4_ (OH)_6_·0.5H_2_O, JCPDS No. 44-0674) [31, 32, 54–57]. Furthermore, a set of tiny peaks at 12.4°, 25.1°, 35.1°, and 41.6° of Zn_4_SO_4_(OH)_6_·0.5H_2_O are observed after discharging to 0.8 V. No other phases, such as ZnO, Mn_2_O_3_, and MnOOH, are present during the discharge/charge processes, confirming the structural stability of the NMOH (Fig. S12). The formation of zinc sulfate hydroxide hydrate is attributed to the increased amount of OH^−^ in the electrolyte due to the insertion of H^+^ to the electrode in the first discharge platform [13, 24, 31]. In subsequent charging process, these new peaks decrease gradually and completely disappear at the end of the charging process, indicating the reversibility of the new phase of Zn_4_SO_4_(OH)_6_·0.5H_2_O.

The surface morphology evolution of the electrode is studied using ex situ SEM images (Fig. 4a). In the early stage of the discharge process, no significant morphology transformation except for the increase in surface roughness was observed. However, after discharging to 1.2 V, the flakes with length and width of several micrometers are generated and maintained stably on the surface of the electrode until their disappearance after recharging to 1.55 V. Simultaneously, the surface morphology of the NMOH electrode remains stable without significant changes. Such a phenomenon is consistent with the XRD results. The EDS mapping images show the uniform distribution of Na, Zn, O, and S elements within the surface of the sample (Fig. S13), further confirming the microflakes to be zinc sulfate hydroxide hydrate. The presented sodium ions are de-intercalated along with the zinc ions upon charging during the first electrochemical cycle and then adsorbed on the surface of the microflakes during the discharge process.

**Fig. 5:**
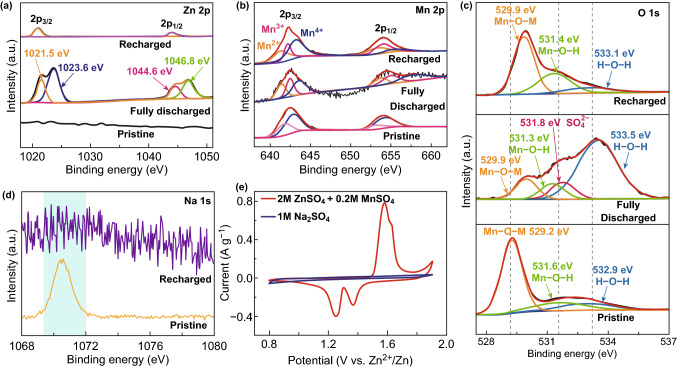
High-resolution XPS spectra of **a** Zn 2*p*, **b** Mn 2*p*, and **c** O 1*s* at initial, fully discharged, and charged statuses. **d** XPS Na 1*s* spectra at different statuses of initial and after recharged to 1.9 V. **e** Cyclic voltammetry curves of the coin-type Zn/NMOH cell at 0.1 mV s^−1^ using aqueous electrolytes of 2 M ZnSO_4_ + 0.2 M MnSO_4_ (red) and 1 M Na_2_SO_4_ (blue). (Color figure online)

To understand the electrode reaction mechanism definitely, the chemical states of the NMOH nanosheets during charge/discharge processes have been characterized (Fig. 5). As illustrated in Fig. 5a, no signal of Zn 2*p* could be detected in the initial state of the NMOH electrode, whereas four peaks are observed after discharge to 0.8 V, demonstrating the insertion of zinc ions into NMOH nanosheets with different coordinations. After recharge to 1.9 V, two peaks disappear, while the intensities of the other two peaks of Zn 2*p* are far below the fully discharged states, confirming that most of zinc ions are extracted from the electrode [20, 30, 31]. Based on the discussion in Fig. 4, the appearance/disappearance of the two peaks at 1023.6 and 1046.8 eV should be ascribed to the reversible formation of zinc sulfate hydroxide hydrate, which is the solid proof for the intercalation of H^+^ into the NMOH, while the other two peaks at 1021.5 and 1044.6 eV are attributed to the intercalation of zinc ions to the NMOH. In particular, the small number of trapped Zn ions in the electrode might serve as the interlayer pillars to further stabilize the layered structure of NMOH upon electrochemical cycles.

As demonstrated above, both H^+^ and Zn^2+^ could insert into the cathode upon discharge, corresponding to a two-step process. Figure S14 shows the EDS patterns of the NMOH electrode and the Zn/Mn molar ratio at different statuses during the second cycle at 200 mA g^−1^. Some Zn^2+^ could be seen in the fully charged cathode with the Zn/Mn molar ratio of 0.13, further certificating the small number of trapped zinc ions in the electrode (Fig. S14a). At the end of the first discharge platform (discharged to 1.3 V), the ratio of Zn/Mn changes to 0.36, indicating that a low quantity of zinc ions insert into the NMOH nanosheets (Fig. S14b). Furthermore, the Zn/Mn ratio increases from 0.36 to 1.05 in the fully charged electrode, proving that the insertion of Zn^2+^ dominates the second discharge process (Fig. S14c). Besides, the ion diffusion coefficients (D) in the first discharge platform (platform I, point 1) and the second discharge platform (platform II, point 2) are investigated with EIS measurements to distinguish the different parts of the discharge platforms (Fig. S15). The calculated ion diffusion coefficient at point 1 (1.36 × 10^−12^ cm^2^ s^−1^) is much larger than that in the point 2 (2.64 × 10^−14^ cm^2^ s^−1^), suggesting that the discharge platforms I and II are essentially ascribed to the insertion of H^+^ and Zn^2+^, respectively, due to the tinier size and electrostatic repulsion of H^+^ than Zn^2+^ [32, 54].

The Mn valence states of the NMOH electrode at different statuses during the discharge/charge processes are shown in Fig. 5b. Different from the pristine sample, the intensities of Mn^3+^ peaks increase after discharged to 0.8 V and Mn^2+^ could be detected, further implying the intercalation of zinc ions into the NMOH sheets and the reduction of Mn^4+^ [15]. In the extraction state, the peak area of Mn^4+^ increases, revealing that most Mn^3+^/Mn^2+^ are reoxidized owing to the extraction of Zn^2+^ from the cathode material [15]. However, the signal of Mn^2+^ does not completely disappear, due to the trapped Zn^2+^ in the electrode. The changes of manganese oxidation state are also characterized by the O 1*s* core-level spectra (Fig. 5c). In addition to the three peaks of Mn–O–M, Mn–O–H and H–O–H in the insertion state, a new peak at 531.8 eV could be assigned to SO_4_^2−^ derived from the new phase of Zn_4_SO_4_(OH)_6_·0.5H_2_O [24, 44]. The intensity of H–O–H peak increases obviously owing to the intercalation of water into the NMOH nanosheets in the form of the water shell around zinc ions. The water molecules are wrapped around the zinc ions, helping reduce the electrostatic effect as well as promoting the transport of zinc ions between the NMOH layers. Furthermore, when the cell recharged to 1.9 V, the peak of SO_4_^2−^ disappears while the intensity of H–O–H peak decreases, which is ascribed to the dissolution of Zn_4_SO_4_(OH)_6_·0.5H_2_O and the extraction of H_2_O. In addition, the peak of Mn–O–M at 529.2 eV moves to a higher binding energy because of the insertion of zinc ions.

Sodium ions also play an important role in the electrochemical process of the NMOH electrode. As shown in Fig. S16, when sodium ions are extracted from the NMOH electrode at first, the electrochemical performance of the electrode is far worse than the electrode that has experienced the discharge process at first, indicating that sodium ions support the NMOH layer for promoting the insertion and transport of zinc ions in the first discharge process. When the cell undergoes discharge process at first, only a broad peak of Na 1*s* could be detected at fully charged state, implying that only a small amount of sodium ions is present in the electrode material (Fig. 5d). Moreover, the results of ICP-AES show that the concentration of sodium ions in the electrolyte increases significantly at the fully charged state (Table S2), meaning that numerous sodium ions are extracted from the electrode during the charging process in the first cycle. Besides, the trapped zinc ions replace sodium ions to form a new phase of Zn_0.13_Na_*x*_Mn_2_O_4_·*n*H_2_O, where the concentration of zinc ions is determined by EDS in Fig. S14. The trapped zinc ions could also serve as the pillar to support the interlayers for subsequent cycles. Such an electrochemical reaction of the NMOH could be classified as the displacement/intercalation reaction mechanism proposed firstly in the current Mn-based cathodes, similar to the reported V-based cathodes [20, 58]. The whole process could be described as follows: During the first cycle, sodium ions act as a pillar for the intercalation of zinc ions upon discharge, because the ionic radius of sodium ions (1.02 Å) is much larger than that of zinc ions (0.74 Å) [18]; furthermore, the zinc ions replace part of sodium ions to stabilize the interlayers of the NMOH, preventing the electrode from collapse and enhancing the performance of the cell during the following discharge/charge process.

Sodium ions not only play a crucial role in cyclic performances of the NMOH cathode, but also contribute to the capacities of the cell. As shown in Figs. 5e and S17, the coin-type Zn/NMOH cell with 1 M NaSO_4_ as the electrolyte delivers a little capacity, because the sodium ions adsorbed on the cathode surface generate the pseudo-capacitance upon discharge [35, 59–61]. The elemental mapping images confirm the presence of adsorbed sodium ions (Fig. S13). The adsorption/desorption mechanism of sodium ions discussed above is consistent with the reported [59]. The concentration of sodium ions in Zn/NMOH (2 M ZnSO_4_ + 0.2 M MnSO_4_) is far below 1 mol L^−1^, and the sodium ions would donate a little capacity for the cell; however, the role of sodium ions is irreplaceable in enhancing the performance of the ZIBs.

**Fig. 6:**
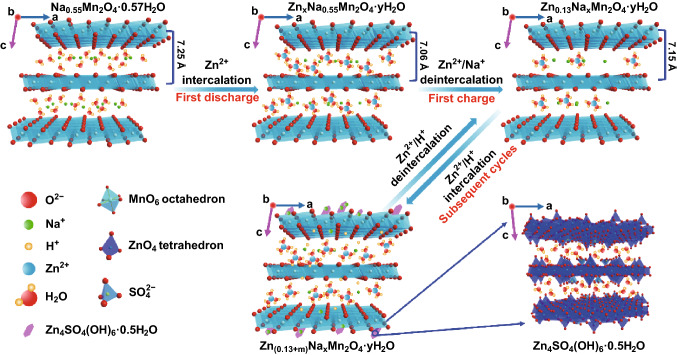
Schematic illustration of the displacement/intercalation reaction mechanism in the first cycle, and the insertion/extraction mechanism of zinc ions in subsequent electrochemical discharge/charge processes

Figure 6 depicts the electrochemical mechanism. In the first discharge process, the solvation zinc ions intercalate into the layered NMOH electrode to replace part of sodium ions that serve as a pillar to support the layered structure, followed by the deintercalation of Zn^2+^/Na^+^ from the NMOH electrode during the first charge process. In the subsequent discharge process, H^+^ insert into the NMOH electrode in the first platform along with the formation of the new phase of Zn_4_SO_4_(OH)_6_·0.5H_2_O, and then, Zn^2+^ insert into the NMOH electrode in the second platform to form the new phase of Zn-inserted phase; meanwhile, sodium ions are adsorbed on the surface to generate a little pseudo-capacitance. In the following charge process, Zn^2+^/H^+^ could extract reversibly from the electrode accompanied by desorption of sodium ions. Such a displacement/intercalation reaction mechanism endows the NMOH electrode with a high reversible capacity and a satisfactory cyclability.

## Conclusions

A layered sodium-ion/crystal water co-intercalated Na_0.55_Mn_2_O_4_·0.57H_2_O was synthesized by the rearrangement of MnO_6_ octahedrons with sodium ions and water molecules via selectively etching the silica tetrahedrons of manganese silicate with NaOH solution. The as-synthesized NMOH with numerous mesopores and active sites is used as the high-performance cathode of ZIBs for the first time. On the basis of the ex situ XPS, XRD, EDS, ICP-AES, and SEM results, a displacement/intercalation mechanism is firstly confirmed in the Mn-based cathode of ZIBs. In a word, the sodium-ion/crystal water co-intercalated NMOH with a large interlayer spacing enables the insertion of zinc ions during the first discharge process, and then a small number of zinc ions replace the sodium ions to improve the stability of the layered structure during the consecutive electrochemical reactions. The reversible insertion/extraction of Zn^2+^/H^+^ into/out of the NMOH is accompanied with the adsorption/desorption of Na^+^ and the reversible formation of Zn_4_SO_4_(OH)_6_·0.5H_2_O during the subsequent discharge/charge cycles. The Na_0.55_Mn_2_O_4_·0.57H_2_O nanosheets show a high reversible capacity of 389.8 mA h g^−1^ at a current density of 200 mA g^−1^, and an excellent cyclability with a high capacity of 201.6 mA h g^−1^ at 500 mA g^−1^ after 400 cycles. Therefore, alkali metal ions and crystal water as the pillar of the Mn-based cathode are effective in stabilizing the crystal structure and improving the transport of zinc ions for high-performance ZIBs.

## Electronic supplementary material

Below is the link to the electronic supplementary material.

Supplementary material 1 (DOC 5458 kb)

## Data Availability

XRD pattern of Mn_*x*_Si_*y*_O_*z*_ and δ-MnO_2_; TG analysis, SEM, and the adsorption isotherm of NMOH; electrochemical characterization of the cell (Zn/NMOH); SEM and corresponding EDS spectra of the NMOH electrode; electrochemical performances of NMOH with different annealing temperatures; comparison of electrochemical properties of NMOH with previously reported Mn-based cathode materials; SEM images of Zn anode at different states; calculated diffusion coefficients of different discharge platforms. The ex situ XRD patterns; the GCD curves and cycling performance with different charging and discharging methods; ICP-AES results of the Na^+^.
