# Supplementary material for: Layered Birnessite Cathode with a Displacement/Intercalation Mechanism for High-Performance Aqueous Zinc-Ion Batteries
Source: Nanomicro Lett. 2020 Feb 18;12:56. doi: 10.1007/s40820-020-0397-3 (PMC7770783; doi:10.1007/s40820-020-0397-3)
Supplement: Supplementary file 1 — Supplementary material 1 (DOC 5458 kb) [file 40820_2020_397_MOESM1_ESM.doc]

**Supporting Information**

Layered Birnessite cathode with a displacement/intercalation mechanism for high-performance aqueous zinc-ion batteries

Xian-Zhi Zhai,^†,‡^ Jin Qu,*^,†^ Shu-Meng Hao,^‡^ Ya-Qiong Jing,^†^ Wei Chang,^‡^ Juan Wang,^†^ Wei Li,^†^ Yasmine Abdelkrim, ^†^ Hongfu Yuan,^†^ Zhong-Zhen Yu*^,†,‡^

^†^ State Key Laboratory of Organic-Inorganic Composites, College of Materials Science and Engineering, Beijing University of Chemical Technology, Beijing 100029, China

^‡^ Beijing Key Laboratory of Advanced Functional Polymer Composites, Beijing University of Chemical Technology, Beijing 100029, China

*E-mails: [qujin@mail.buct.edu.cn](mailto:qujin@mail.buct.edu.cn) (J. Qu); [yuzz@mail.buct.edu.cn](mailto:yuzz@mail.buct.edu.cn) (Z.-Z. Yu)


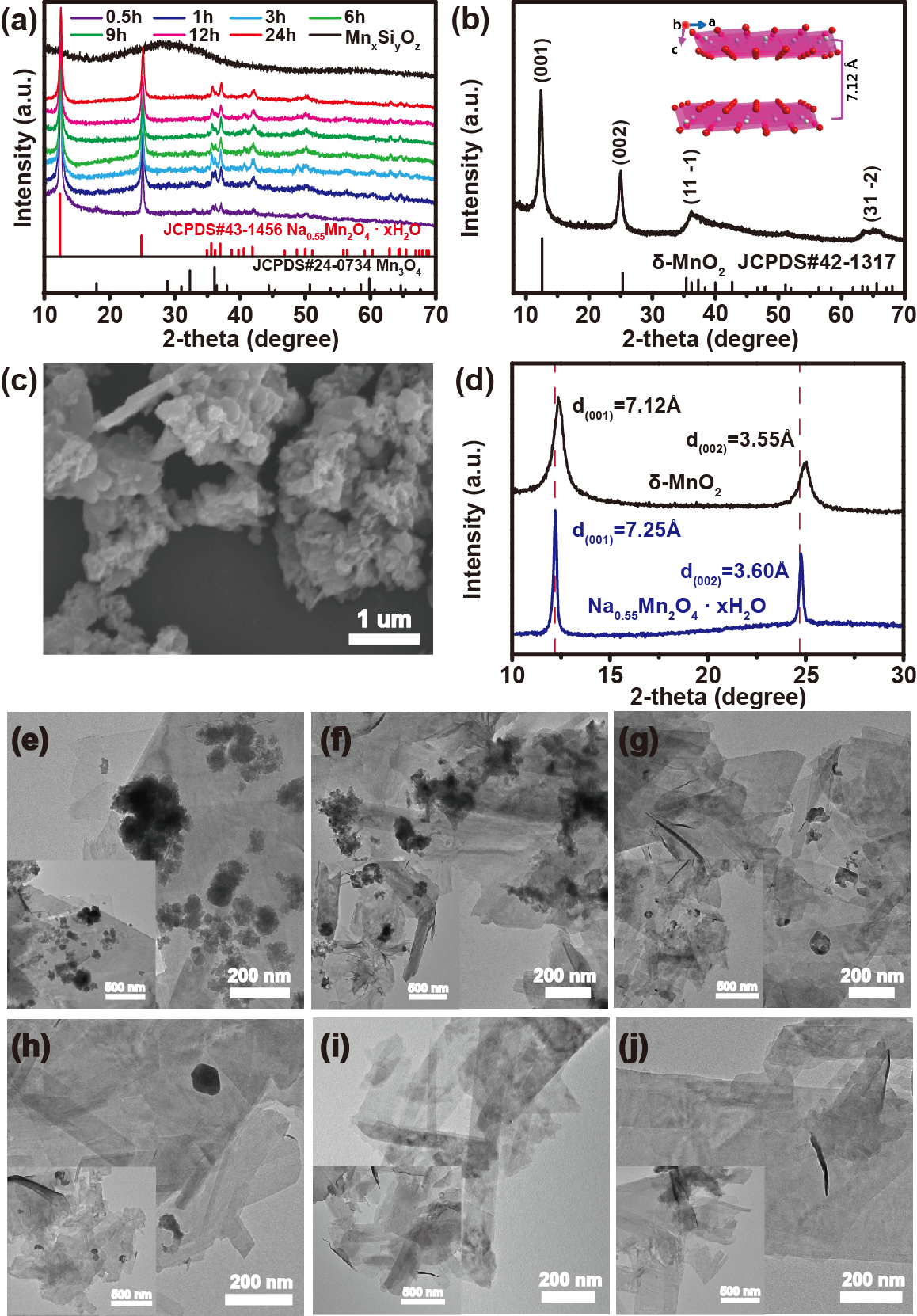


**Fig. S1.** (a) XRD patterns of the Mn_x_Si_y_O_z_ and NMOH with different etching time. (b) XRD pattern of the δ-MnO_2_ and an illustration of its crystal structure (inset). (c) SEM image of δ-MnO_2_. (d) XRD patterns of NMOH and δ-MnO_2_. The TEM images of NMOH with different etching time: (e) 1h, (f) 3h, (g) 6h, (h) 9h, (i) 12h, (j)24h.


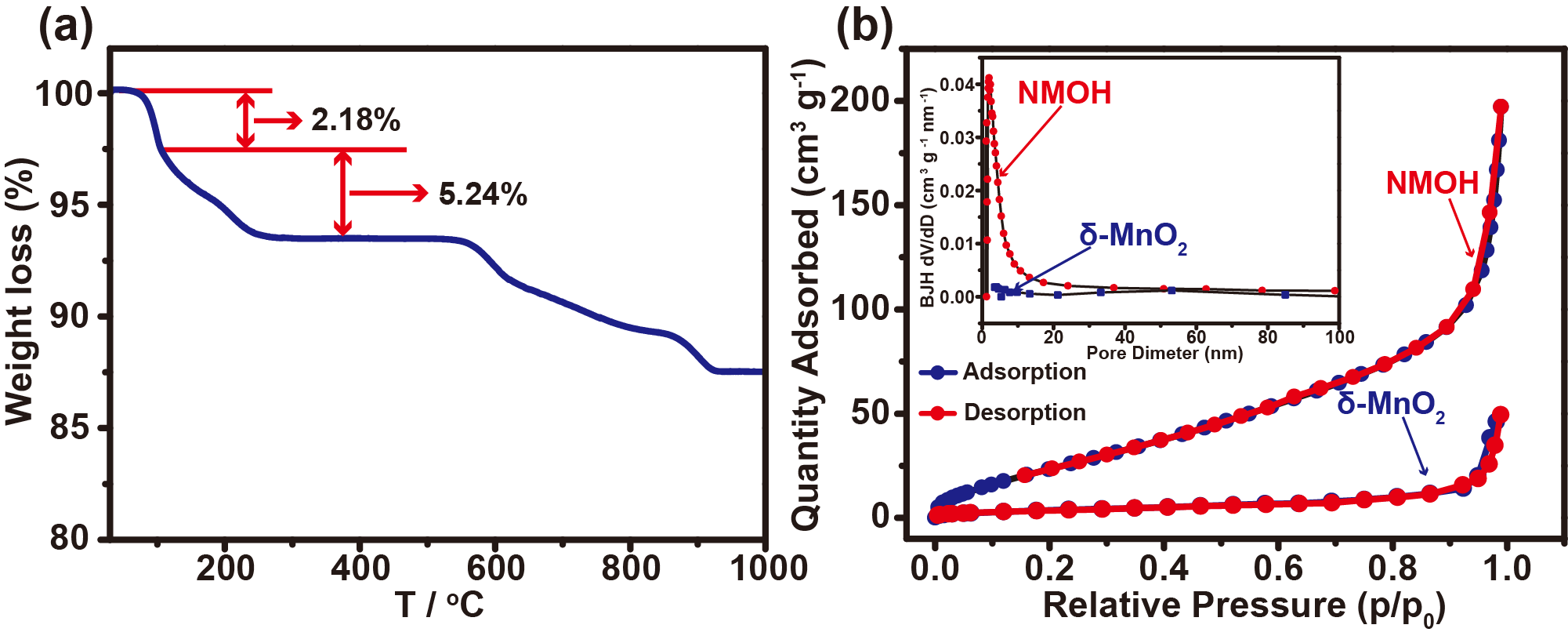


**Fig. S2**. (a) TGA analysis and (b) Nitrogen adsorption-desorption isotherms of NMOH and δ-MnO_2_. The inset is the pore-size distribution calculated using the BJH method (Red: NMOH; Blue: δ-MnO_2_). The δ-MnO_2_ has a low BET specific surface areas of 13.456 m^2^ g^-1^.


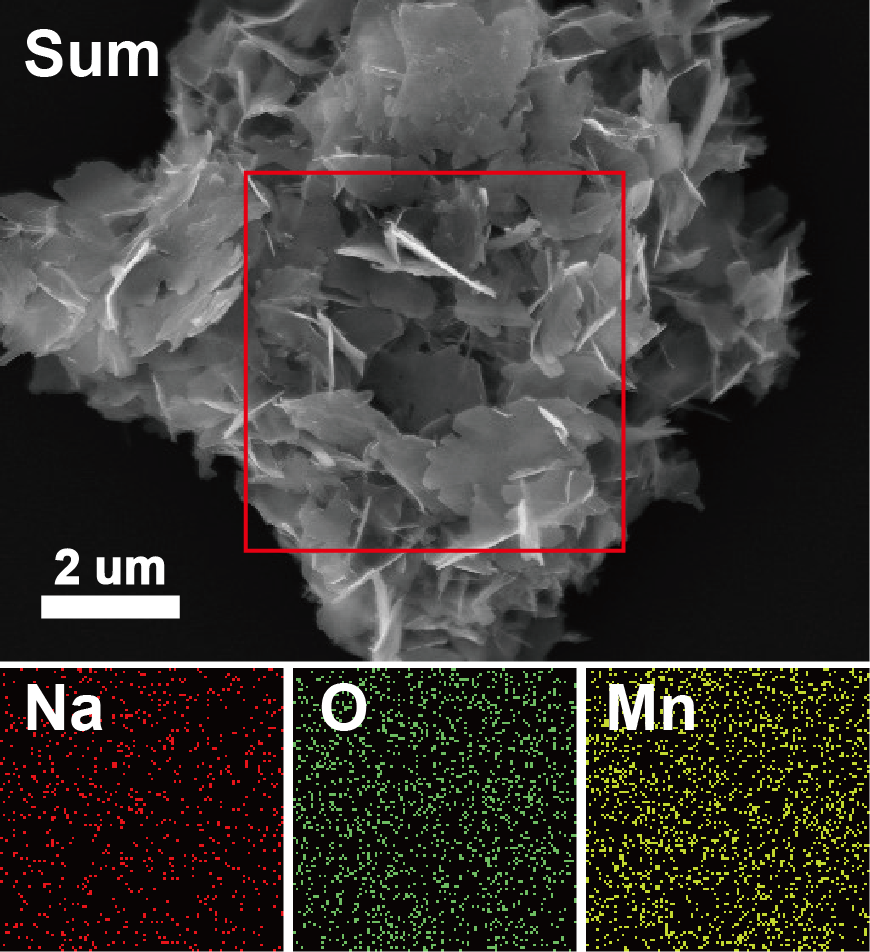


**Fig. S3.** SEM image and corresponding elemental mapping images of NMOH.


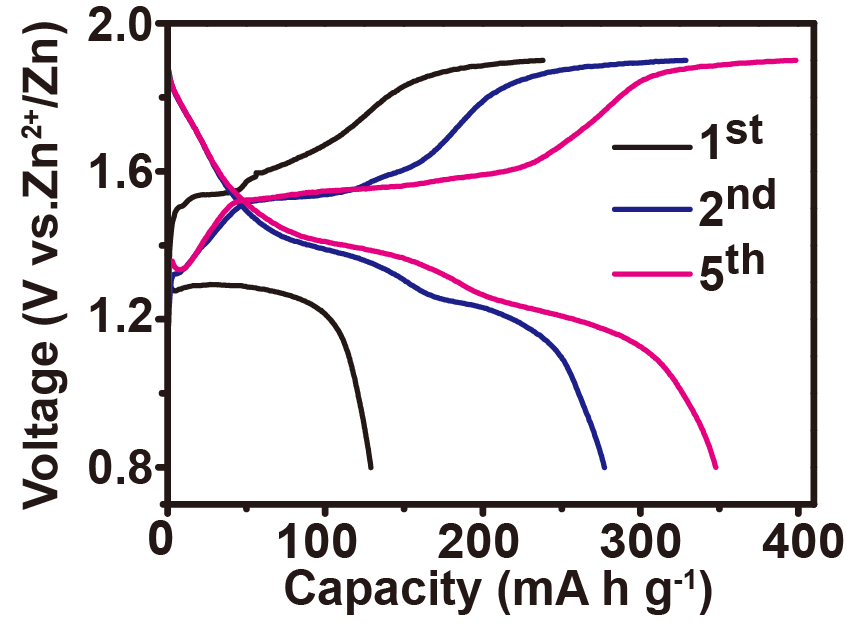


**Fig. S4.** Galvanostatic charge/discharge profiles of the Zn/NMOH cell tested at a current density of 200 mA g^-1^.


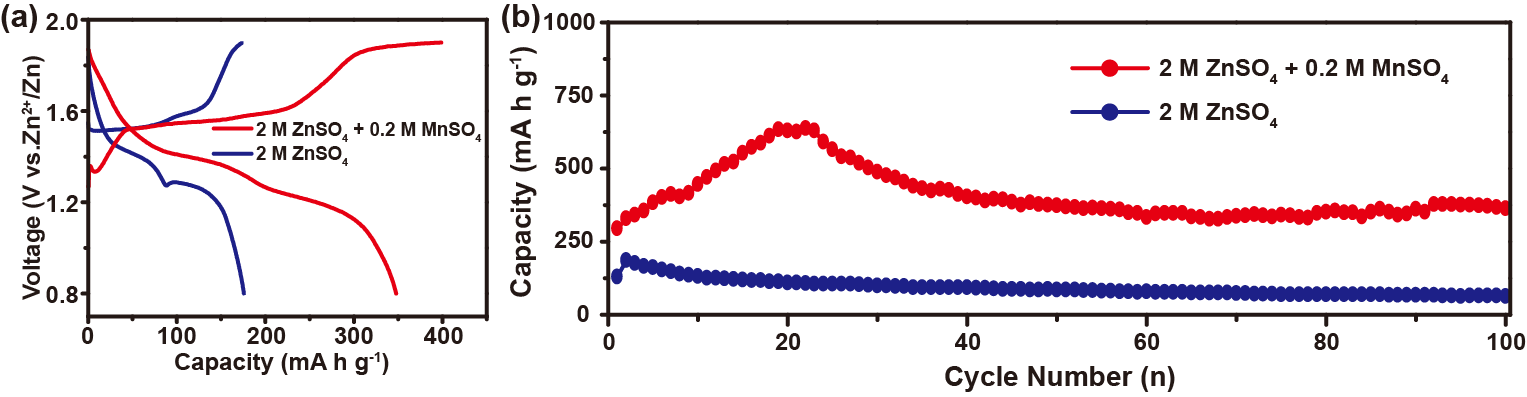


**Fig. S5.** (a) Galvanostatic charge/discharge profiles and (b) cycling performances of the coin-type Zn/NMOH cell at the current density of 200 mA g^-1^ using an aqueous electrolyte of 2 M ZnSO_4_ with and without the 0.2 M MnSO_4_ additive.


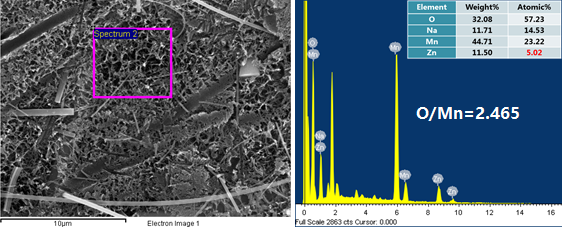


**Fig. S6.** SEM image and corresponding EDS spectrum of NMOH cathode at fully charged state.


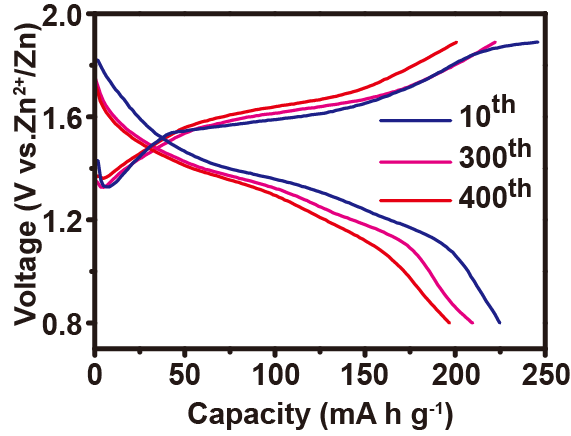


**Fig. S7.** Galvanostatic charge/discharge curves of NMOH cathode at 500 mA g^-1^ between 0.8 and 1.9 V.


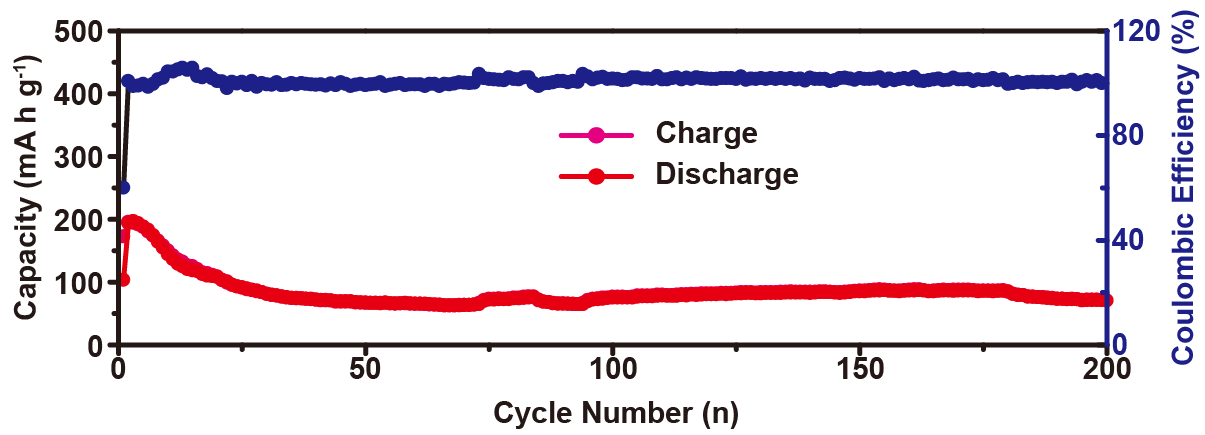


**Fig. S8.** Cycling performances of δ-MnO_2_ electrode using an aqueous electrolyte of 2 M ZnSO_4_ + 0.2 M MnSO_4_ at a current density of 500 mA g^-1^.


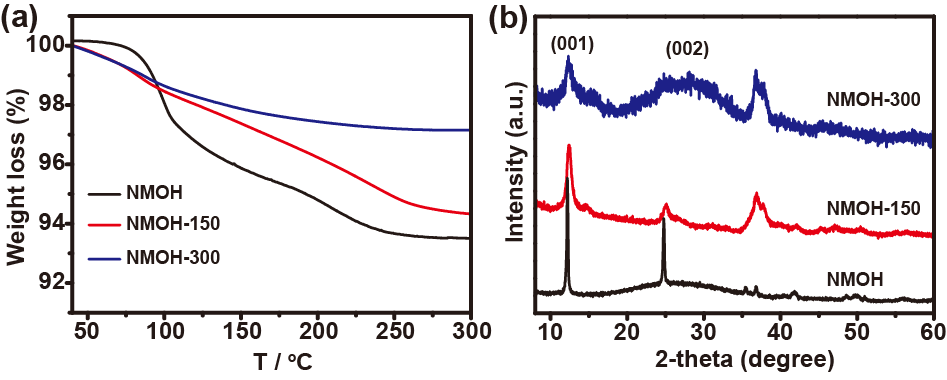


**Fig. S9.** (a) TGA curves and (b) XRD patterns of NMOH at different thermal treatment temperatures.


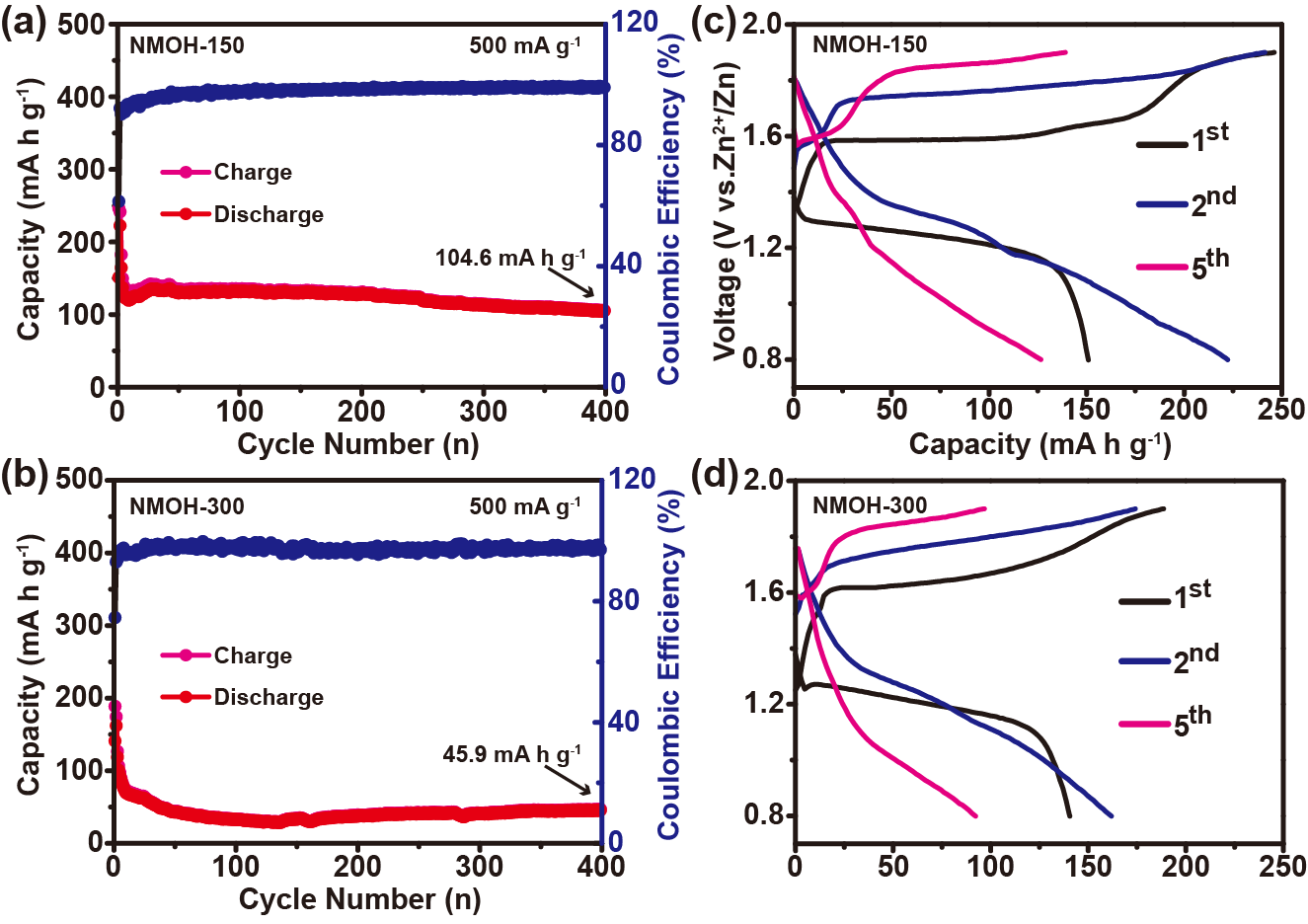


**Fig. S10.** Cycling performances of (a) NMOH-150, (b) NMOH-300 and corresponding Galvanostatic charge/discharge curves of (c) NMOH-150, (d) NMOH-300 at the current density of 500 mA g^-1^ using an aqueous electrolyte of 2 M ZnSO_4_ with the 0.2 M MnSO_4_ additive.


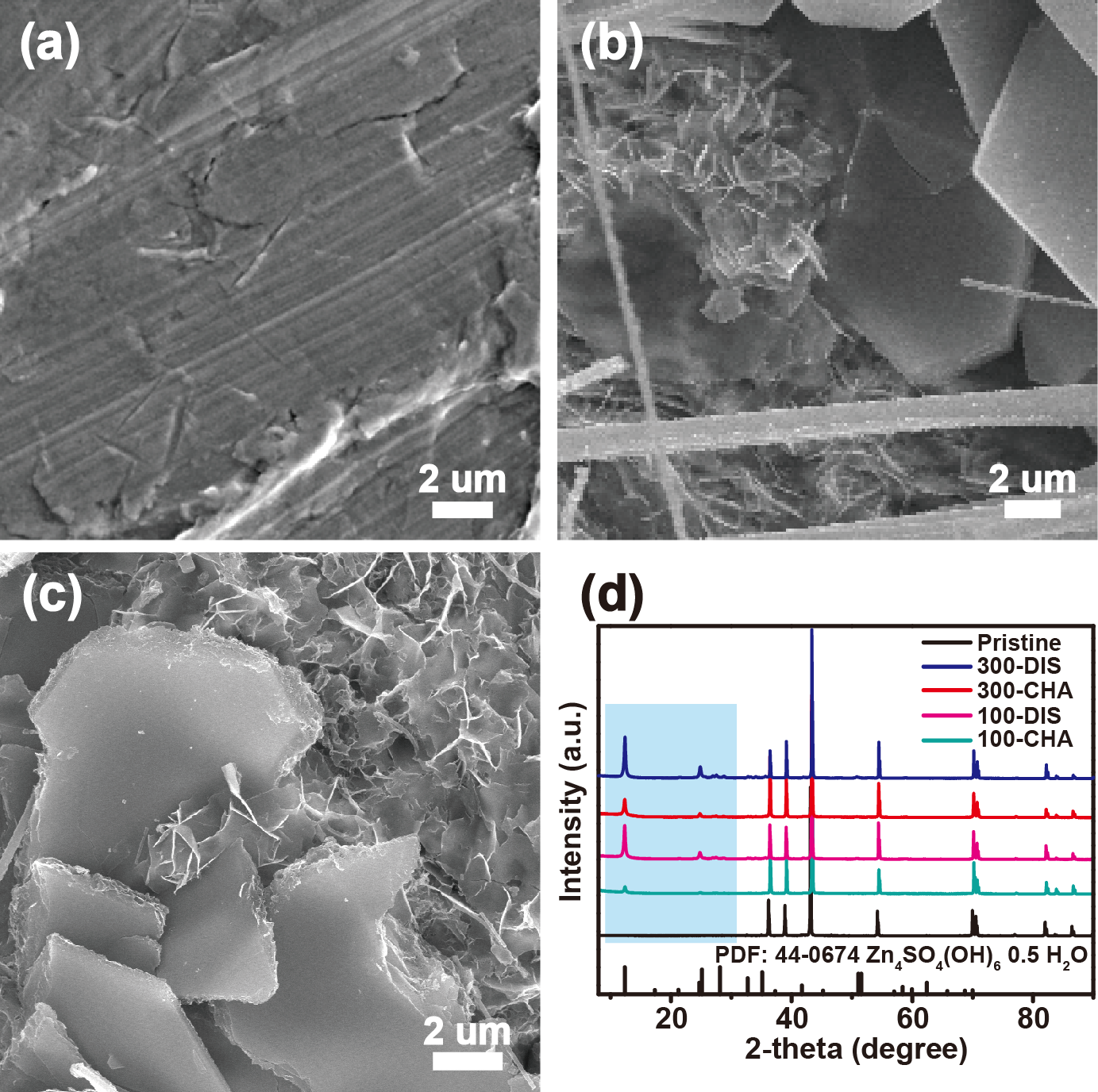


**Fig. S11.** SEM images of Zn anode at different states: (a) pristine, (b) after 100 cycles, and (c) after 300 cycles. (d) XRD patterns of Zn anode in different cycles. As depicted in Fig. S11 (a-c), the new phase maintained in the Zn anode after 100 cycles. And the microflake should be indexed to the zinc sulfate hydroxide hydrate (Zn_4_SO_4_ (OH)_6_·0.5H_2_O) which will affect the deposition of zinc ions, resulting in the decrease of capacity.


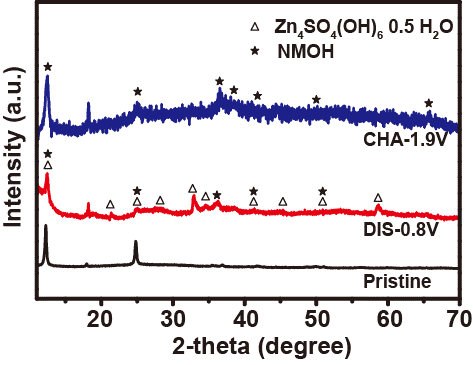


**Fig. S12.** XRD patterns of the NMOH cathode at different charge/discharge states.


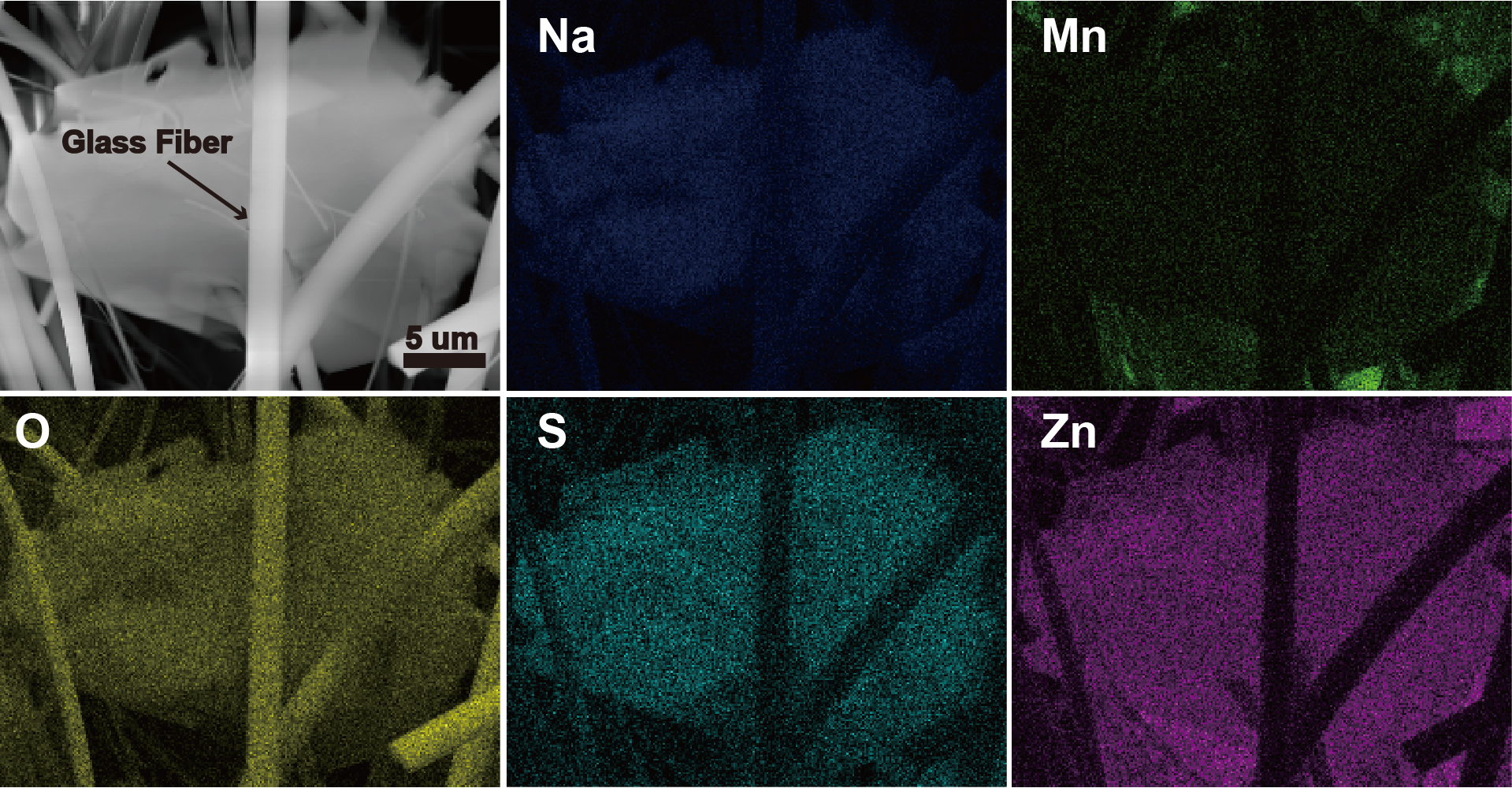


**Fig. S13.** SEM and elemental mapping images of the new phase at fully discharged state. The fiber-like materials in these images are glass fibers from the separator.


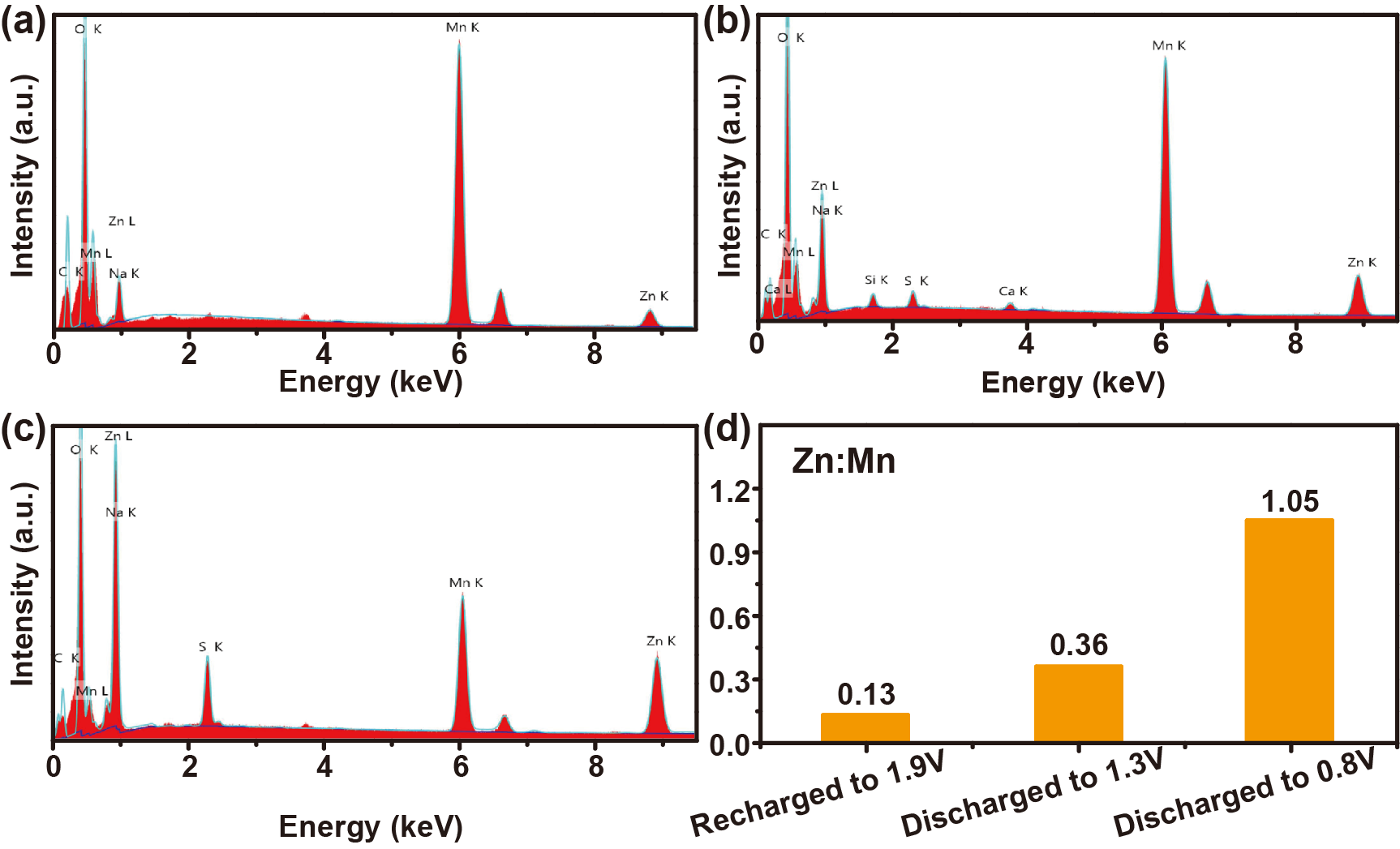


**Fig. S14.** EDS spectra of the NMOH electrode during the second cycle at 200 mA g^-1^: (a) charged to 1.9 V, (b) discharged to 1.3 V, and (c) discharged to 0.8 V. (d) Zn/Mn ratios in the electrode at different statuses. Note: the zinc in the ZHS phase is excluded when calculating the ratio of Zn/Mn.


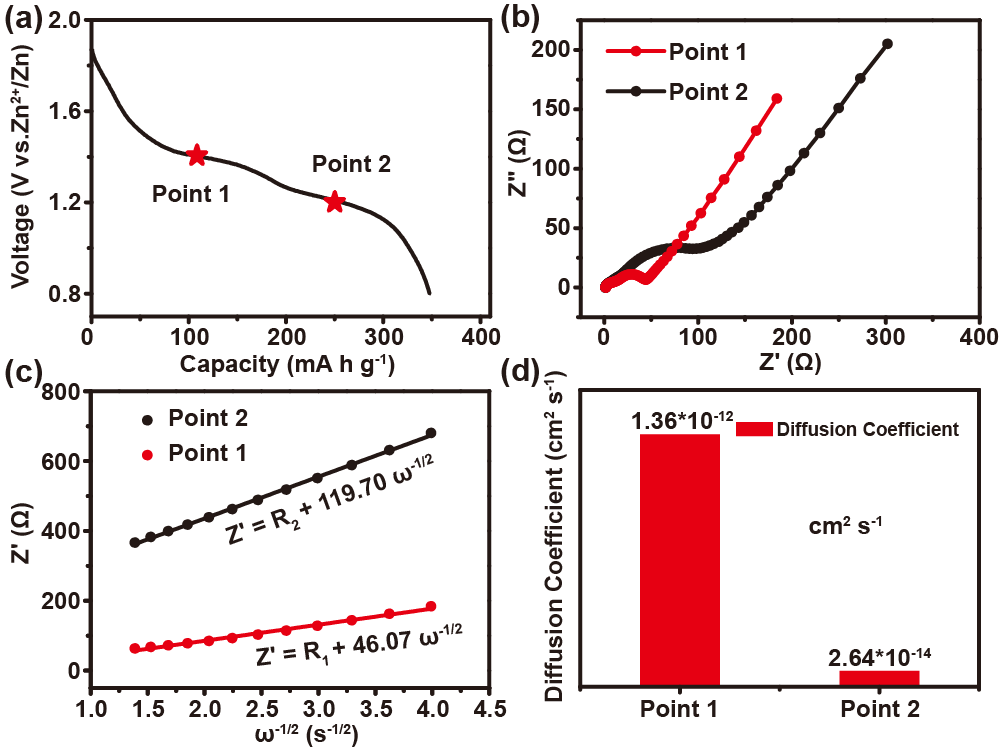


**Fig. S15.** (a) Typical discharge profile of the Zn/NMOH cell. The points 1 and 2 are collected for EIS test. (b) Nyquist spectra of the cells at the points 1 and 2. (c) Z’ vs. ω^−1/2^ plots of NMOH electrode in the low frequency region. (d) Calculated diffusion coefficients of different discharge platforms. The diffusion coefficient of the Zn/NMOH cell is calculated using the following equation[1]:

$D=\frac{R^{2}T^{2}}{2A^{2}n^{4}F^{4}C^{2}\sigma^{2}}$

Where *R* is the gas constant, *T* is the experiment temperature, *n* is the electron number per molecule participating the redox reaction, *F* is the Faraday constant, *A* is the surface area of the electrode, *C* is the molar concentration of insertion ions in NMOH electrode, and σ is the Warburg coefficient calculated from the Z’ vs. ω^−1/2^ plots.


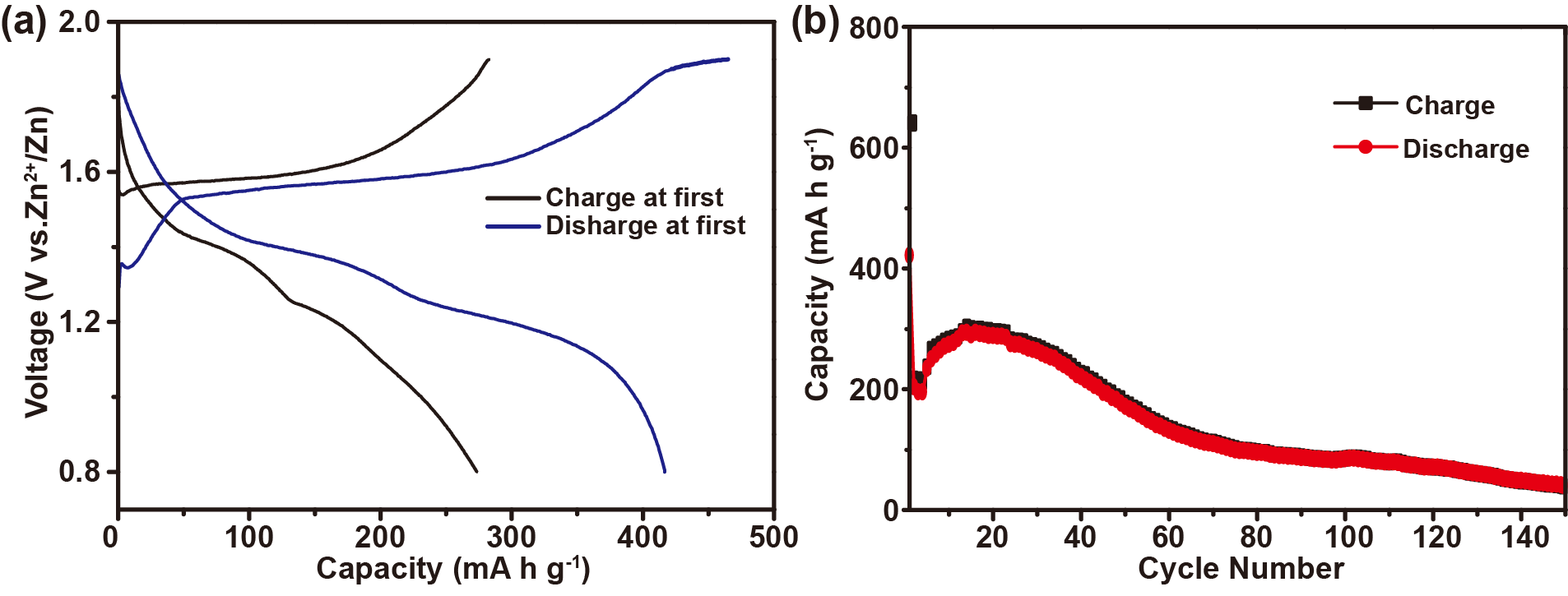


**Fig. S16.** (a) The 10^th^ galvanostatic charge/discharge curves of the NMOH cathode with different charge and discharge methods at 200 mA g^-1^ between 0.8 and 1.9 V. (b) Cycling performances of the NMOH cathode which charges at first at a current density of 100 mA g^-1^.


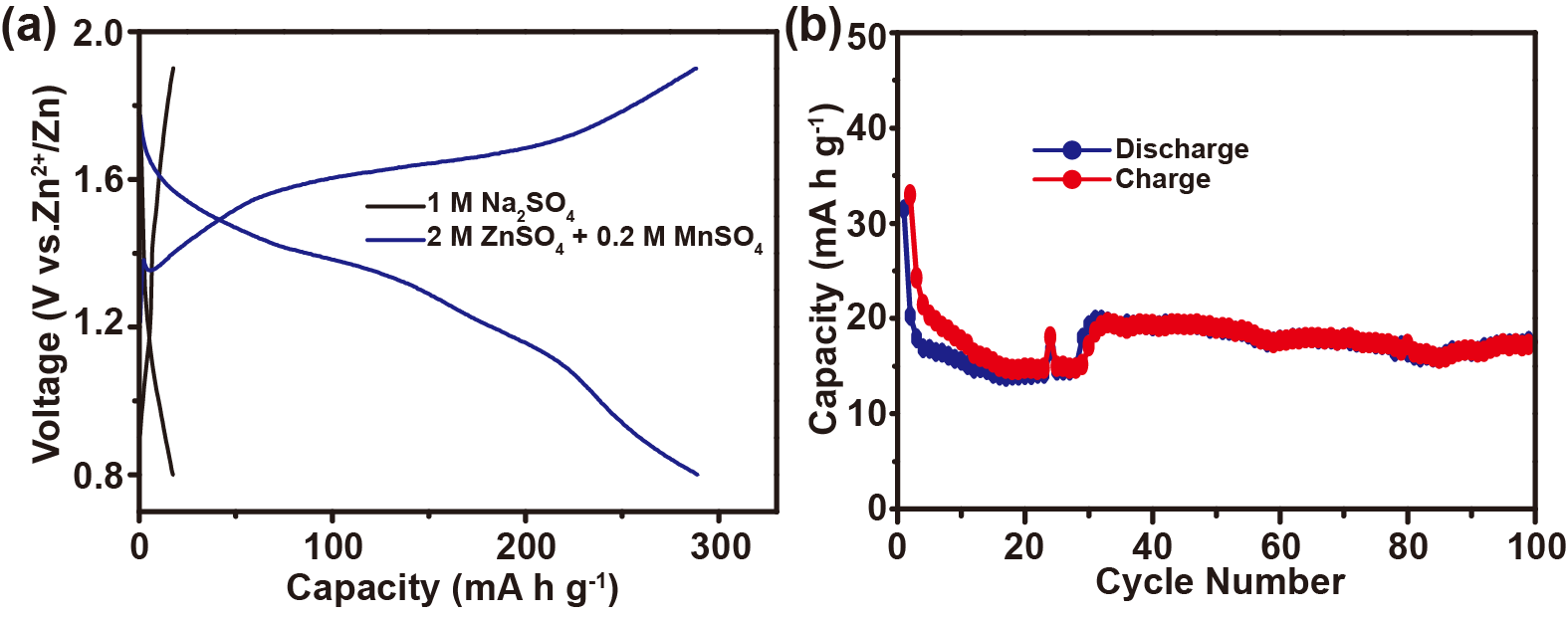


**Fig. S17.** (a) Typical galvanostatic charge/discharge curves with different electrolytes at 200 mA g^-1^ between 0.8 and 1.9 V of the Zn/NMOH cell. (b) Cycling performances of the Zn/NMOH cell using 1M Na_2_SO_4_ aqueous electrolyte at a current density of 200 mA g^-1^.

**Table S1.** Comparison of electrochemical properties of NMOH with other Mn-based cathode materials reported in the literature.

| Cathode Materials | Electrolytes | Specific Capacity/Rate Performance | Refs. |
| --- | --- | --- | --- |
| Na_0.55_Mn_2_O_4_ ·0.57 H_2_O | 2 M ZnSO_4_+0.2 M MnSO_4_ | 389.8 mA h g^-1^ at 200 mA g^-1^  87.1 mA h g^-1^ at 1500 mA g^-1^ | **This work** |
| MgMn_2_O_4_ | 1M MgSO_4_ + 1M ZnSO_4_ + 0.1M MnSO_4_ | 269 mA h g^-1^ at 100 mA g^-1^  58 mA h g^-1^ at 2400 mA g^-1^ | [2] |
| MnO_2_ | 1 M ZnSO_4_ | 350 mA h g^-1^ at 100 mA g^-1^  - | [3] |
| ZnMn_2_O_4_/N-doped graphene | 1 M ZnSO_4_+0.05 M MnSO_4_ | 221 mA h g^-1^ at 100 mA g^-1^  75 mA h g^-1^ at 2000 mA g^-1^ | [4] |
| β-MnO_2_ | 1 M ZnSO_4_ | 270 mA h g^-1^ at 100 mA g^-1^  86 mA h g^-1^ at 1056 mA g^-1^ | [5] |
| Todorokite-MnO_2_ | 1 M ZnSO_4_ | 108 mA h g^-1^ at 50 mA g^-1^  - | [6] |
| Spinel Mn_3_O_4_ | 2 M ZnSO_4_ | 239 mA h g^-1^ at 100 mA g^-1^  51.8% retained at 2000 mA g^-1^ | [7] |
| MnO_2_ on carbon fiber paper | 2 M ZnSO_4_+0.2 M MnSO_4_ | 290 mA h g^-1^ at 90 mA g^-1^  58.6% retained at 1950 mA g^-1^ | [8] |
| CuHCF | 1 M ZnSO_4_ | 56 mA h g^-1^ at 20 mA g^-1^  66.3% retained at 288 mA g^-1^ | [9] |
| Spinel ZnMn_2_O_4_@C | 3 M Zn(CF_3_SO_3_)_2_ | 150 mA h g^-1^ at 50 mA g^-1^  80 mA h g^-1^ at 500 mA g^-1^ | [10] |
| ZnHCF@MnO_2_ | 0.5 M ZnSO_4_ | 118 mA h g^-1^ at 100 mA g^-1^  75.2 mA h g^-1^ at 1000 mA g^-1^ | [11] |
| ZnMn_2_O_4_@PEDOT | 1 M ZnSO_4_ | 221 mA h g^-1^ at 0.5 mA cm^-2^  62.5 mA h g^-1^ at 10 mA cm^-2^ (1.66 A g^-1^) | [12] |
| ZnMn_2_O_4_ | 1 M ZnSO_4_+0.5 M MnSO_4_ | 106.5 mA h g^-1^ at 100 mA g^-1^  70.2 mA h g^-1^ at 3200 mA g^-1^. | [13] |
| (PANI)-intercalated MnO_2_ | 2 M ZnSO_4_+0.1 M MnSO_4_ | 280 mA h g^-1^ at 200 mA g^-1^  110 mA h g^-1^ at 3000 mA g^-1^ | [1] |

**Table S2.** ICP-AES results of the sodium ions in 2 M ZnSO_4_ + 0.2 M MnSO_4_ electrolytes before and after 2 cycles.

| Different status | Na^+^ (ppm) |
| --- | --- |
| Pristine | 6.165 |
| Two cycle | 9.148 |

**References**
